# Rescue of hematopoietic stem/progenitor cells formation in *plcg1* zebrafish mutant

**DOI:** 10.1038/s41598-018-36338-8

**Published:** 2019-01-21

**Authors:** Karine F. Ferri-Lagneau, Jamil Haider, Shengmin Sang, TinChung Leung

**Affiliations:** 1 The Biomedical/Biotechnology Research Institute, North Carolina Central University, North Carolina Research Campus, Nutrition Research Building, Kannapolis, NC 28081 USA; 2 Laboratory for Functional Foods and Human Health, Center for Excellence in Post-Harvest Technologies, North Carolina A&T State University, North Carolina Research Campus, Nutrition Research Building, Kannapolis, NC 28081 USA; 3 Department of Biological & Biomedical Sciences, North Carolina Central University, Durham, NC 27707 USA

## Abstract

Hematopoietic stem/progenitor cells (HSPC) in zebrafish emerge from the aortic hemogenic endothelium (HE) and migrate towards the caudal hematopoietic tissue (CHT), where they expand and differentiate during definitive hematopoiesis. Phospholipase C gamma 1 (Plcγ1) has been implicated for hematopoiesis *in vivo* and *in vitro* and is also required to drive arterial and HSPC formation. Genetic mutation in *plcg1*^−/−^ (*y10* allele) completely disrupts the aortic blood flow, specification of arterial fate, and HSPC formation in zebrafish embryos. We previously demonstrated that ginger treatment promoted definitive hematopoiesis via Bmp signaling. In this paper, we focus on HSPC development in *plcg1*^−/−^ mutants and show that ginger/10-gingerol (10-G) can rescue the expression of arterial and HSPC markers in the HE and CHT in *plcg1*^−/−^ mutant embryos. We demonstrate that ginger can induce *scl/runx1* expression, and that rescued HE fate is dependent on Bmp and Notch. Bmp and Notch are known to regulate nitric oxide (NO) production and NO can induce hematopoietic stem cell fate. We show that ginger produces a robust up-regulation of NO. Taken together, we suggest in this paper that Bmp, Notch and NO are potential players that mediate the effect of ginger/10-G for rescuing the genetic defects in blood vessel specification and HSPC formation in *plcg1*^−/−^ mutants. Understanding the molecular mechanisms of HSPC development *in vivo* is critical for understanding HSPC expansion, which will have a positive impact in regenerative medicine.

## Introduction

During vascular development, endothelial progenitors give rise to a network of blood vessels including arteries and veins. Arterial specification, differentiation and morphogenesis are orchestrated by evolutionarily conserved signaling pathways including vascular endothelial growth factor (Vegf), Notch and ephrinB2^1,2^. The establishment of arterial identity is also a prerequisite for the emergence of definitive hematopoietic stem/progenitor cells (HSPC). Therefore, it is imperative to understand the role of critical genes in the differentiation and specification of arteries and the development of definitive HSPCs. Phospholipase C gamma 1 (Plcγ1) function is required downstream of Vegf receptors (Vegfr1 and Vegfr2) to drive arterial specification and HSPC development during vertebrate embryogenesis^3,4^. Plcγ1 has been implicated for hematopoiesis *in vivo* and differentiation of embryonic stem cells into erythrocytes and monocytes/macrophages *in vitro*^5^ for classical T-cell receptor signaling and T-cell activation^6^, and for early B-cell development^7^. In the developing embryo, expression of Vegf-a is induced by sonic hedgehog (Shh) to establish midline-to-lateral and dorsal-ventral gradients from the hypochord and the ventral part of somites; thus, axial angioblasts are patterned to adopt the arterial fate, while lateral angioblasts receiving a smaller amount of Vegf stimulation become endothelial cells (ECs) with a venous identity^8^. In addition, a strong gradient of Vegf-a binds to Vegf- and neuropilin- coreceptor complexes (Vegfr2-Nrp1) of arterial differentiating ECs, mediated through Plcγ1 to activate the Raf1-Mek-Erk cascade. As a result, Vegf-a activates Notch signaling to promote arterial cell fate via ephrinB2. Notch signaling is required for the leading endothelial tip cells’ migration and the connection to the pre-existing artery to form a functional vascular network^9^. On the other hand, a lower gradient of Vegf-a promotes venous EC differentiation. The venous signal activates the PI3K-Akt1 pathway which inhibits the Plcγ1-induced Raf1 signaling and promotes expression of venous markers such as ephrinB4, or COUP-TFII, which also inhibits ephrinB2 expression^10,11^. Arterial-venous identity is specified when hemodynamic force of blood flow is being established; interestingly, manipulation of blood flow could override the genetic programming of arterial-venous specification and interchange arteries and veins^12^.

Hematopoiesis and vasculogenesis are closely linked to each other. As in mammals, hematopoiesis in zebrafish is characterized by two waves, primitive and definitive hematopoiesis. Definitive HSPCs arise along the ventral wall of the dorsal aorta (DA) from the presumptive hemogenic endothelium (HE), undergoing endothelial-to-hematopoietic transition (EHT). During definitive hematopoiesis, zebrafish HSPCs undergo EHT around 30 to 35 hour-post-fertilization (hpf)^13^ from the luminal side of the dorsal aorta. These cells directly emerge from aortic endothelium^13,14^ and migrate through the sub-aortic space^13^ to the caudal hematopoietic tissue (CHT). Some of these cells could enter the circulation through the axial vein, then extravasate at widely diverse locations and migrate through the mesenchyme to reach the thymus^15^. Others could migrate from the CHT to kidney marrow, before matured cells go into circulation^13^. Scl was identified as a marker of the HE during the EHT process^16–18^. The emerging HSPCs express *scl, myb* and *runx1* and enter the circulation to home transiently to the CHT, where they could multiply and differentiate from 2 to 7 days-post-fertilization (dpf), prior to seeding their permanent hematopoietic organs^19,20^. Like other stem cell niches, the CHT is associated with a vascular bed, the caudal vascular plexus (CVP), characterized by large sinusoids in which the reduced flow of blood progenitors helps the homing process at the CHT^20^. The CVP also provides a microenvironment for interaction of the developing HSPCs with secreted factors and cytokines necessary for the HSPCs to be instructed and to differentiate^15,21,22^. In this hematopoietic microenvironment, HSPCs undergo extensive proliferation and further migrate to seed the definitive hematopoietic organs, the thymus and kidney marrow, giving rise to many blood lineages^20,23^. Therefore, understanding the molecular mechanisms of HSPC development *in vivo* is critical for HSPCs expansion, which will have a positive impact in regenerative medicine.

Bmp signaling acts specifically on the definitive hematopoietic program to induce HSPC emergence within the HE of the DA^24^. Scl is required for the development of the DA^16,25^ and promotes EHT in the HE downstream of Shh and Notch, and up-stream of Runx1^8^. Yet, Scl and Myb play important roles in EHT and migration of HSPCs to the CHT^26,27^, and Notch is required for arterial specification^28^. *plcg1*^−/−^ zebrafish embryos are defective in both arterial morphogenesis and HE specification^3^. This mutant provides the opportunity to investigate the relationship of hemogenic endothelium and HSPC development.

Based on the fact that the EHT process is dependent on Notch^13,14,29–31^, both Bmp and Notch can regulate NO production and play potential roles in enhancing hematopoiesis^32^. Thus, we ask if the effect of ginger on induced *scl/runx1* expression for the rescued HE fate is dependent on Bmp and/or Notch. We also investigate whether NO plays any role in the rescue of the HSPC fate in *plcg1*^−/−^ zebrafish embryos. Here, we show that ginger treatment can rescue the deficiencies of *plcg1*^−/−^ zebrafish embryos in HE and HSPC development, and partially restores the blood circulation. We also demonstrate that ginger can dramatically increase the production of NO independent of nitric oxide synthase (Nos) functions. Using a NO scavenger, we show that ginger-induced NO production is required for rescuing the expression of arterial and HSPC markers and blood circulation.

In summary, ginger/10-G treatment can induce NO and potential players in Bmp, Notch and NO pathways, which are required for the rescue of the genetic defects in blood vessel specification and HSPC formation in *plcg1*^−/−^ zebrafish embryos, thus providing a foundation for future study of signaling crosstalk for promoting hematopoiesis.

## Results

### Ginger rescues the DA and HSPC in *plcg1*^−/−^ embryos

**Figure 1:**
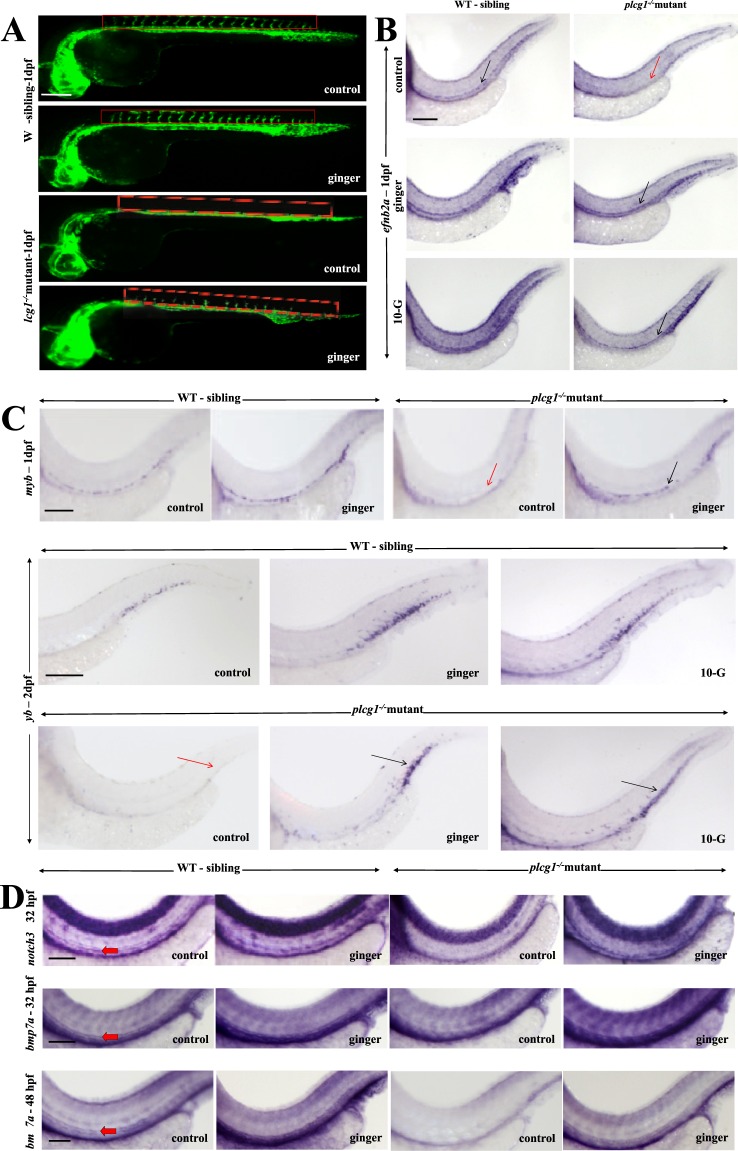
Ginger rescues the dorsal aorta (DA) and the formation of hematopoietic stem/progenitor cells (HSPCs) in *plcg1*^−/−^ embryos. (**A**) Real-time imaging of the vasculature in *tg(fli1:GFP)* embryos at 30 hpf. Red rectangle shows the location of ISV. (**B**) *In situ* hybridization of the DA marker ephrin-B2a (*efnb2a)* at 1dpf (32 hpf). Black arrow indicates the artery, red arrow shows absence of artery in mutant fish. (**C**) *In situ* hybridization of the HSPC marker *myb* at 1 (32 hpf) vs 2dpf (54hpf). Black arrow points to *myb* expression in hemogenic endothelium (1 dpf) and CHT region (2 dpf), red arrow indicates absence of expression in mutant fish. (**D**) *In situ* hybridization of *notch3* (normally expressed in the DA at 1 dpf (32 hpf)) and *bmp7a* (shown here for the first time to be expressed in the DA from 1(32 hpf) to 2 dpf (48 hpf)). These marker expressions are lost in *plcg1*^−/−^ embryos and rescued following exposure to ginger. Red arrow points to DA. Scale bars = 250 μm.

Zebrafish with the *plcg1*^−/−^ recessive mutation showed impaired arterial-venous vasculogenesis^3^ and cardiac ventricular contractility^33^. Similar to *dead beat*^33^ (another lethal allele of *plcg1*), here, the *y10* allele^3^ homozygous mutation completely abolished Plcγ1 function, resulting in the absence of arteries, HSPCs and blood circulation^3,34^. No arterial-venous specification is found in *plcg1*^−/−^ embryos, with these embryos harboring only a single axial cord with no lumen at 1dpf (Fig. 1A). In vertebrates, Plcγ1 controls arterial morphogenesis downstream of Vegf signaling^3^. We use the transgenic and mutant zebrafish line *tg(fli1:EGFP)*;*plcg1*^−/−^ (*y10* allele)^3^ to visualize the developing vasculature, sort homozygous mutants from their wildtype (WT) siblings (Fig. 1A), and study the effect of ginger/10-G on their compromised definitive hematopoiesis. Surprisingly, real-time observation of the fluorescent vessels reveals a partial rescue (intersegmental vessel, ISV formation in 17.5% embryos) of the vasculature in arterial-venous morphogenesis at 1dpf by ginger/10-G treatment (Fig. 1A). This is done by exposure of *plcg1*^−/−^ embryos to ginger (or its active compound 10-G) from the late gastrulation (9–10 hpf) stage onward^35^. This also reveals an increase of vasculature in the CHT region in ginger-treated WT sibling embryos. Ginger (83%) and 10-G (89.5%) rescue the formation of the DA at 32 hpf, as shown by the restored expression of the arterial marker *efnb2a*, which is not expressed in *plcg1*^−/−^ embryos (Fig. 1B). Circulation is also partially rescued in some of the *plcg1*^−/−^ mutants (17.5%) at 2 dpf (data not shown). This also rescues the expression of the HSPC marker *myb* along the aortic HE, and later in the CHT at 2 dpf stage (Fig. 1C) of mutants, suggesting the rescue of definitive hematopoiesis. We choose two different timings using the *myb* marker because around 1 dpf, the *myb* + cells originally emerge along the DA, and around 2 dpf, they migrate to the CHT region. Notch signaling is necessary for arterial cell-fate specification during vasculogenesis as part of the Vegf-Notch-ephrinB2a signaling cascade and for HSPC specification in the HE^36^. *In situ* hybridization analysis confirms the rescue of arterial identity in *plcg1*^−/−^ embryos treated with ginger by using the arterial marker *notch3* which is absent in the mutants (Fig. 1D). Further supporting the above finding, we demonstrate that *bmp7a* is also expressed in the DA of WT siblings at 1–2 dpf stage (but not in *plcg1*^−/−^ embryos); *bmp7a* expression is also rescued in the restored DA of ginger-treated *plcg1*^−/−^embryos (Fig. 1D).

These data indicate a robust effect of ginger in rescuing the *plcg1*^−/−^ embryos in specification of arterial fate and HSPC formation; however, ginger treatment is not sufficient to rescue the mutant embryos from a severe heart dysfunction. On the other hand, there is no significant change in the expression of the vein marker *fms-*related tyrosine kinase 4 (*flt4*) in ginger-treated *plcg1*^−/−^ embryos; however, a rescue of lumenization of the cardinal vein is observed (Supplemental Fig. S1).

### Bmp and Notch signaling in ginger-induced arteriogenesis and HSPC formation

**Figure 2:**
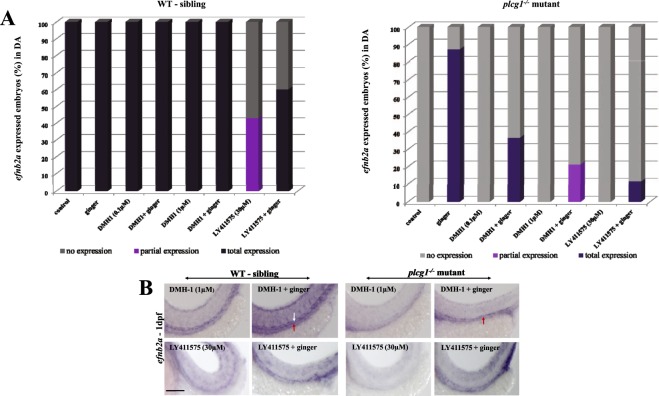
Signaling pathways involved in the rescue of arteriogenesis by ginger in *plcg1*^−/−^ embryos. (**A**) Pharmacological inhibition of Bmp (DMH-1) and Notch (LY411575) signaling pathways affect the rescue of dorsal aorta, analyzed by *efnb2a in situ* at 1 dpf. (**B**) Representative images of different treatment. White arrow indicates the dorsal aorta, underneath is the vein indicated by red arrow. Scale bars = 250 μm.

**Figure 3:**
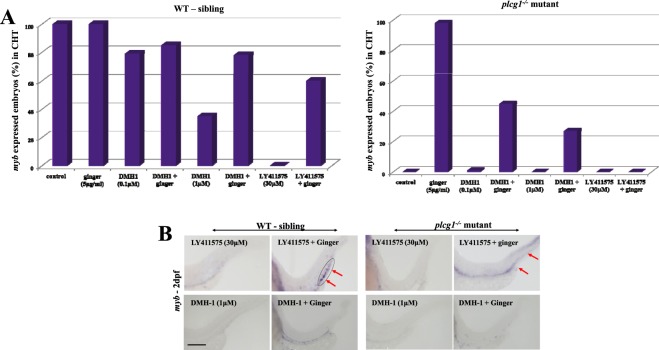
Signaling pathways involved in the rescue of definitive hematopoiesis by ginger in *plcg1*^−/−^ embryos. (**A**) Pharmacological inhibition of Bmp (DMH-1) and Notch (LY411575) signaling pathways affect the rescued HSPCs, analyzed by *myb in situ* at 2 dpf. (**B**) Representative images of different treatment. Encircled area indicates *myb* expression in the CHT region, whereas red arrows indicate the CHT region only. Scale bars = 250 μm.

We previously demonstrated that ginger promoted definitive hematopoiesis via Bmp signaling in zebrafish embryos^35^. We showed that treatment with a highly selective Bmp inhibitor DMH-1, which had no effect on arterial morphogenesis, inhibited *myb* expression in the CHT of WT embryos. Our finding was supported by Wilkinson and colleagues’ demonstration that the arterial program was unaffected by loss of Bmp signaling, but the latter was required for HSPC emergence within the aortic HE^24^. Here, we show ginger can rescue both arteriogenesis (Fig. 2A,B) and hematopoiesis (Fig. 3A,B) in *plcg1*^−/−^ embryos, which are dependent on an intact and functional Bmp signaling.

Since the process of definitive hematopoiesis occurs later during development, we cannot use a knockdown approach such as antisense morpholino to inhibit genetic pathways in which morpholinos are normally delivered by microinjection into the 1-cell stage embryos, or a knockout approach, because both will interfere with the signaling pathways during gastrulation. Therefore, we choose a chemical-genetic approach using chemical inhibitors to modulate signaling pathways after the gastrulation stage. Inhibitors can be applied to the embryo medium at the end of gastrulation (9–10hpf) to study the signaling of ginger-induced arteriogenesis and hematopoiesis. Since Plcγ1 is already a downstream signal of the Vegf pathway for arterial and HSPCs development during vertebrate embryogenesis^3^, here, we intend to study other downstream players of potential interacting pathways during the rescue of HSPC formation in the *plcg1*^−/−^ embryos.

Notch signaling was downstream of Shh and Vegf pathways and was essential, but not sufficient for inducing arterial identity (*efnb2a*)^37,38^, although it was crucial for HSPC generation from the HE^39^. Figure 2A,B shows Notch signaling is required for rescuing specification of the DA in *plcg1*^−/−^ mutants after ginger treatment. For HSPCs, treating WT siblings with the γ-secretase inhibitor LY411575 (Notch inhibitor) abolishes *myb* expression in the CHT, whereas 60% of embryos co-exposed to LY411575 and ginger show a rescue of *myb*-expressing cells in their CHT. In *plcg1*^−/−^ embryos, Notch inhibition abolishes the effect of ginger on the rescue of HSPC formation in the CHT (*myb*) (Fig. 3A,B). Taken together, Notch signal is required for the rescue effect of ginger on the DA specification and HSPC formation in the *plcg1*^−/−^ mutant. These findings provide preliminary steps for future study of signaling crosstalk between ginger-induced Bmp and Notch pathways in hematopoiesis.

### Ginger expands the CVP at the perivascular niche

**Figure 4:**
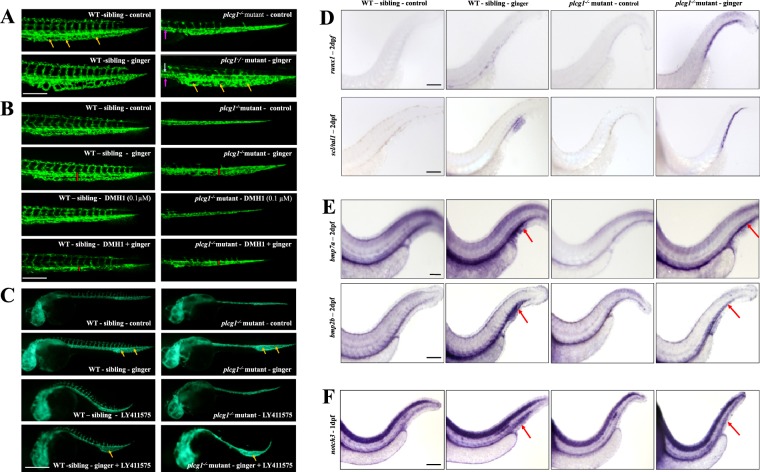
Ginger expands the Caudal Vein Plexus (CVP). (**A**) Ginger increases or rescues the CVP sinusoids in *tg(fli1:GFP)* WT siblings or *plcg1*^−/−^ mutant embryos respectively (2 dpf). Yellow arrows show enlarged CVP with big sinusoid. White arrow indicates dorsal aorta and pink arrow cardinal vein. (**B**) Bmp is necessary for the effect of ginger on the CVP in *tg(fli1:GFP)* WT siblings and *plcg1*^−/−^ mutant embryos (2 dpf). Vertical red bar represents the width of CVP. (**C**) Inhibition of Notch compromised the effect of ginger on the developing CVP at the sinusoidal level (1 dpf). Yellow arrows show enlarged CVP with big sinusoid. (**D**) *runx1* and *scl* are ectopically expressed in the CVP/CHT area following ginger treatment. (**E**,**F**) *Bmp7a/bmp2b* expression (**E**) and *notch3* expression (**F**), are highly promoted by ginger in the CVP/CHT of WT and *plcg1*^−/−^mutant embryos. Red arrow indicates high expression in CVP/CHT (**E**,**F**). Pictures are the representative images of ≥30 embryos per condition. Red box indicates the CHT region. Scale bars = 250 μm.

The formation of the CVP involved active sprouting, ventral migration of endothelial cells and anastomosis at 1 dpf, then maturation and lumenization of the primordial CVP to allow blood circulation at 2 dpf^20,40^. Treating WT siblings with ginger results in enlarged CVP sinusoids (Fig. 4A). This might help HSPC homing and docking to the CHT associated with such vascular beds. Ginger-treated *plcg1*^−/−^ embryos harbor a rescued axial aorta from the trunk to the CVP region and rescued sinusoids within the CVP (Fig. 4A). These rescue effects are dependent on Bmp, when we incubate these embryos with the Bmp inhibitor DMH-1 (0.1 µM), the rescue effect of ginger is diminished (Fig. 4B).

Notch is also an important signal for the development of the perivascular niche and the regulation of HSC emergence from the hemogenic endothelium^41^. Embryos exposed to the Notch inhibitor LY411575 do not develop a CVP; co-exposure to ginger reverses the effect and results in an expansion of CVP in WT siblings and mutant embryos (Fig. 4C). Since HSC fate is established by the Notch–Runx pathway^39^, and Notch and Scl promote the embryonic endothelial-to-hematopoietic transition^17^, we want to investigate the effect of ginger on *scl* and *runx1* in the rescue of the *plcg1*^−/−^ embryos. It is known that *scl* plays a role in the specification of the HE^18^, and *runx1* specifically orchestrates HSPC trans-differentiation from the HE via EHT^13^. *scl* is then required for HSPC maintenance^18^, and *myb* is required for the migration of these HSPCs towards the CHT^26^. Here, we show there is no expression of *scl* and *runx1* at 2 dpf in the CHT of WT siblings and *plcg1*^−/−^ embryos (Fig. 4D). Interestingly, ginger treatment to WT siblings can induce a weak expression of *runx1* and an over-expression of *scl* in the CHT (and tail fin region), while *plcg1*^−/−^ mutants treated with ginger show strong ectopic expression of both *scl* and *runx1* in the ventral tail portion, along the vascular structure of the CHT. These results suggest that ginger might have the potential to induce transcription factors like *scl* and *runx1* to possibly activate the HE and EHT activities in the developing CHT of *plcg1*^−/−^ embryos. This raises an interesting question: do the rescued embryos have the capability to utilize the CHT as a novel site for HE and HSPC development in response to ginger treatment?

We cannot directly confirm if the expanded CHT becomes a novel site for HE and HSPC development, but we do observe that ginger induces expression of *bmp7a*/*bmp2b* and *notch3* in the CHT of WT siblings and mutant embryos (Fig. 4E,F). The arterial marker *efnb2a* and HSPC marker *myb* are also over-expressed in the CHT/CVP of ginger-treated embryos (Fig. 1B,C). To further confirm that ginger induces more HSPCs in the CHT, blood lineage markers are evaluated by whole-mount *in situ*. Indeed, erythroid (*gata1)*, early myeloid *(pu.1*), neutrophil *(mpo)*, myeloid (*lcp1*), and lymphoid (pan-leukocyte *cd45*) cell fates are all promoted by ginger (Supplemental Fig. S2).

### Ginger induces nitric oxide (NO) production throughout the embryo

**Figure 5:**
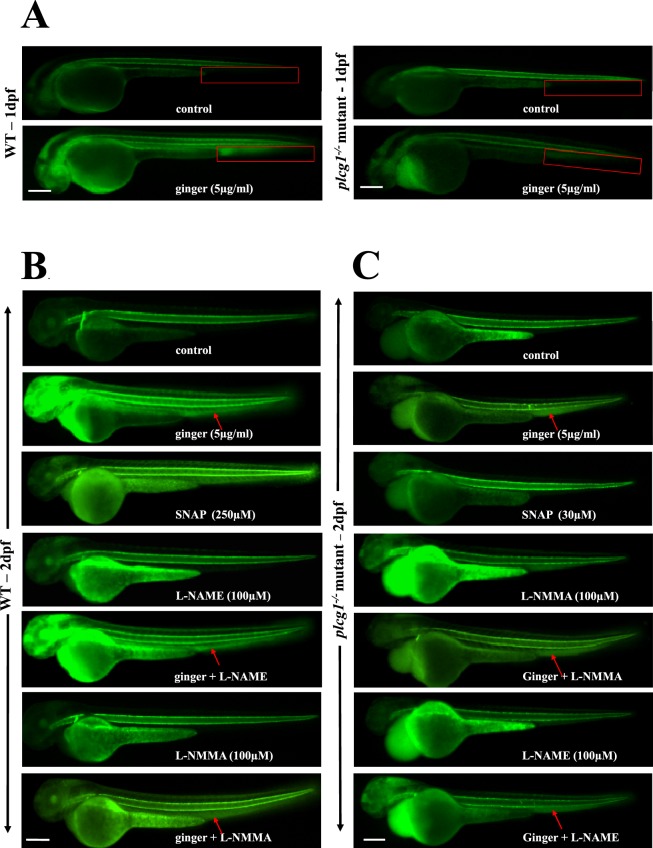
Ginger increases the level of nitric oxide (NO) in non-transgenic WT and *plcg1*^−/−^ mutant embryos. (**A)** Effect of ginger on NO staining at 1 dpf WT and *plcg1*^−/−^ embryos. (**B**,**C**) Effect of NO donor (SNAP) and 2 Nos inhibitors (L-NAME, L-NMMA) on 2 dpf WT (**B**) and *plcg1*^−/−^ embryos (**C**). Red arrows show ginger effect in CVP region. The effect of the NO donor SNAP is shown for comparison. Non-transgenic WT and mutant embryos are used to see the NO fluorescent dye. Pericardial edema, a typical phenotype occurred in *plcg1*^−/−^ mutants at 2 dpf (**C**). Pictures are the representative images of ≥30 embryos per condition. Scale bars = 250 μm.

Vegf signaling can regulate NO via its downstream effectors Plcγ1 and Nos in endothelial cells. NO is a short-lived gaseous intercellular signal that regulates many important functions such as arteriogenesis, cardiovascular homeostasis, vascular tone and micro-vascular permeability. NO was required for blood flow and definitive hematopoiesis^32^, and was the most consistent mediator of collateral arteriogenesis in mammals^42^ and in zebrafish^43^. For NO detection *in vivo*, we use the diaminofluorescein-FM diacetate (DAF-FM-DA) probe, a sensitive fluorescent dye, for detecting the physiological range of NO concentrations. WT control embryos produce NO in the notochord from 1 dpf onwards (Fig. 5A) and in the cleithrum from 2 dpf (Fig. 5B), which is consistent with Lepiller *et al*.^44^. Upon exposure to ginger (from late gastrulation onwards, or following a 30-minute incubation at 2 dpf), embryos harbor a tremendous, ectopic increase of the NO levels in the heart, head, notochord, vessels/blood, and CHT, which can be completely inhibited by co-incubation with the NO scavenger c-PTIO (400 μM, 30 mins incubation) (Supplemental Fig. S3A). On the other hand, inhibition of nitric oxide synthases by L-NMMA or L-NAME shows no effect on the NO-promoting action of ginger in WT embryos (Fig. 5B). Using the NO donor SNAP as a positive control, we observe increased levels of NO in the notochord and caudal fin, but not in the head and heart. Most interestingly, ginger treatment has a greater effect on NO production than SNAP (30 uM) in the zebrafish embryos. SNAP and c-PTIO data support the finding that ginger induces NO production (Fig. 5B,C, Supplemental Fig. S3A). This effect of ginger on NO production is also observed in *plcg1*^−/−^ embryos, especially in the CVP/CHT and the circulation. The Nos inhibitors have no dramatic effect on the ginger-induced increase of NO production in the CHT region (Fig. 5B,C). As an additional control, sodium nitrite (NaNO_2_; 1–2 mM) (Supplemental Fig. S3B) is able to induce NO levels with the same pattern as observed in WT and *plcg1*^−/−^ SNAP-treated embryos (Fig. 5B,C). However, the *tg(fli1:EGFP);plcg1*^−/−^ embryos in the same experimental conditions show no effect of NaNO_2_ on the CVP (Supplemental Fig. S3C), which look like the CVP of control *plcg1*^−/−^ mutants (with no sinusoids), in contrast to the effect of ginger on these mutant embryos (Fig. 4A).

### Ginger-induced NO production is essential for arteriogenesis and hematopoiesis

**Figure 6:**
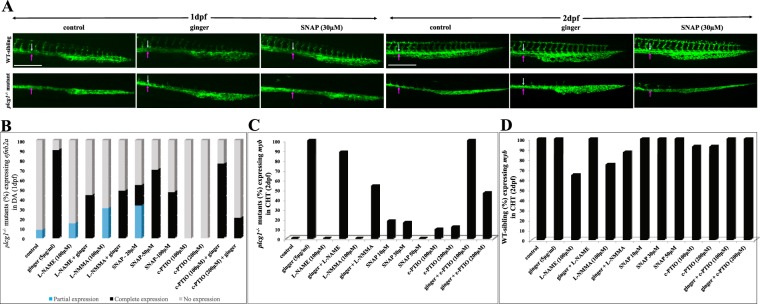
Effects of nitric oxide on arterial and hematopoietic gene marker expression. (**A**) Vasculature visualized by *tg(fli1:GFP)* at 1 dpf and 2 dpf embryos. (**B**–**D**) The graphical results are shown in 1 dpf (**B**) and 2 dpf (**C**,**D**) embryos. NO synthase inhibitors L-NAME and L-NMMA, NO donor SNAP, and NO scavenger c-PTIO. White arrow indicates dorsal aorta and pink arrow indicates cardinal vein. Scale bars = 250 μm.

Co-incubation with ginger and the Nos inhibitors L-NAME or L-NMMA lead to a 50%-decrease in the number of rescued *plcg1*^−/−^ mutant embryos showing expression of *efnb2a* in the DA; exposures to SNAP at various concentrations rescue *efnb2a* expression in the DA in 70% of *plcg1*^−/−^ embryos (compare with 90% rescued by ginger); c-PTIO can abolish the ginger-induced rescue of DA specification (Fig. 6A,B). The rescue of defective arterial ECs in SNAP-treated *plcg1*^−/−^ embryos is observed at 1 dpf stage (Fig. 6A,B). In contrast to ginger, SNAP does not rescue the NO signal in the CVP region, the morphogenesis of the CVP nor definitive HSPC proliferation at 2 dpf (Figs 5C and 6C). In the same experimental series, L-NAME slightly inhibits ginger-induced rescue of HSPCs in *plcg1*^−/−^ mutants, whereas L-NMMA and c-PTIO inhibit ginger effect by about 50% (Fig. 6C). Figure 6D shows the effects of the same treatments on hematopoiesis in WT siblings: the Nos inhibitor L-NAME inhibits about 30% of the number of WT siblings with HSPCs in their CHT. This partial inhibition is reversed when co-treated with ginger, whereas L-NMMA inhibits about 25% of the number of embryos with definitive hematopoiesis (a 13.6% is reversed when co-incubated with ginger). In contrast to what is observed in *plcg1*^−/−^ mutants, c-PTIO combined with ginger does not affect hematopoiesis in WT embryos (Fig. 6C,D).

In addition, Notch inhibitor (LY411575) does not reverse the increase in NO observed in the ventral tail region following ginger treatment, but it does promote the ginger-induced NO production in the CVP/CHT region (Supplemental Fig. S4A). The Bmp inhibitor DMH-1 decreases the control levels of NO production *in vivo* at 2 dpf, but only partially reverses the effect induced by ginger; in both cases, the slight decrease in NO is observed only in the notochord (Supplemental Fig. S4B). Consistently, Bmp inhibitor DMH-1 decreases NO production in the notochord and caudal fin, and slightly reverses ginger-induced increase of NO in the notochord and in all regions of the 3 dpf embryos (Supplemental Fig. S4C). Thus, the NO stimulating effect of ginger, which is sustained and widely distributed throughout the embryos (compared to SNAP), might be at least partially required for rescuing the defects in arteriogenesis and definitive hematopoiesis of *plcg1*^−/−^ mutants.

## Discussion

Development of the endothelial and hematopoietic lineages has long been considered to be (EHT) closely related processes. Back in 1917, Sabin suggested that some hematopoietic cells were derived from hematogenous yolk sac endothelial cells in the developing chick embryo^45^. Murray then named these multipotent mesenchyme-derived cells, common to endothelium and blood, as “hemangioblasts”^46^. Murray was the first to suggest that there was also a specific endothelium retaining the capacity to give rise to new blood cells. “Hemogenic endothelium” (HE) was observed in real-time *in vivo* using the transparent zebrafish embryo; Bertrand *et al*., showed the emergence of *flk1*^+^*/myb*^+^ cells from the ventral wall of the DA, as well as the presence of these markers in adult differentiated blood cells^14^. At the same time, Kissa and Herbomel also described the trans-differentiation of endothelial-like cells into hematopoietic precursors through a maturation process, called the “endothelial hematopoietic transition” (EHT)^13^. In zebrafish, some of these HE-derived definitive HSPCs could migrate to the bilateral thymi, while others transiently reside at the posterior CHT to expand before seeding the kidney marrows^47^. We show that ginger, a natural product, can rescue both defects of the vasculogenesis of the DA and the formation of HSPCs in the CHT in *plcg1*^−/−^ mutants. These effects are dependent on the pathways involved in the control of aortic specification and definitive hematopoiesis such as Bmp/Notch/Scl/Myb. In addition, ginger treatment dramatically increases the level of NO overall, particularly in the axial vasculature, CHT, heart and brain. Blocking the activity of the Nos by inhibitors does not dramatically reduce the ginger-induced NO production (Fig. 5B,C); however, it does reduce about 50% of ginger-induced rescue of DA (Fig. 6B) and HSPCs (Fig. 6C) in *plcg1*^−/−^ embryos. In contrast to the Nos inhibitors, the NO scavenger c-PTIO is able to completely abolish the effect of ginger on the induced-production of NO (Supplemental Fig. S3A). Consistent with these results, c-PTIO is also more efficient than Nos inhibitors for blocking the effect of ginger for rescuing arteriogenesis and hematopoiesis in *plcg1*^−/−^ embryos (Fig. 6B,C). NO is a small lipophilic, uncharged, and short-lived molecule that can cross the plasma membranes^48^. Endogenous NO is formed by both enzymatic (Nos) and non-enzymatic reactions in the cardiovascular system. Nitrate and especially nitrite in the blood and extra vascular tissues could be reduced to form NO^49^. In normal conditions, beside Nos, other enzymes and compounds can generate NO via nitrite reductase activity. These enzymes and compounds include xanthine oxidoreductase, aldehyde oxidase, hemoglobin, deoxy-hemoglobin, myoglobin, carbonic anhydrase, mitochondrial enzymes of the transport chain, glutathione-S-transferases, cytochrome P450, carbonic anhydrase, vitamin C, antioxidants, and polyphenols^50,51^. Thus, the ginger-induced production of NO might be related to the ginger-induced increase in erythrocytes^35^, as well as to its antioxidant pathways^52–54^. These data are consistent with our observations and the well-known properties of ginger. Moreover, North *et al*., also demonstrated that NO was a conserved regulator of HSPC development and functioned downstream of Notch for arterial identity and HSPC induction^32^. This agrees with our data, suggesting that the ginger-induced increase in NO production could compensate the Notch signaling pathway as it pertains to arteriogenesis. Interestingly, NO is required for the ginger-induced rescue of arteriogenesis and hematopoiesis in *plcg1*^−/−^ embryos, and the NO staining pattern in the notochord (immediately above the developing DA) and surrounding area around the CHT, suggesting that it could provide direct inductive signals for DA formation.

This study provides a foundation that intervention such as ginger treatment could rescue the hematopoiesis defect in *plcg1*^−/−^ mutant embryos. It raises an unanswered but interesting question: can interventions such as induction by ginger/Bmp be used to manipulate a highly vascular region like CVP to become a favorable microenvironment to support the nurture or proliferation of HSPC in zebrafish, as well as the bone marrow niche in human? Future study is needed to evaluate if it is possible that the zebrafish CVP can be induced to become a secondary site for EHT for the emergence of HSPC from the endothelium, or if *in vitro* culture of human bone marrow cells can be manipulated to support HSPC expansion.

Our study of the rescue of genetic defects in HSPC formation may provide insight to understanding the genetic basis induced by interventions such as with ginger. It may also indicate a future path of a new experimental paradigm to regulate hematopoiesis, such as our previous study on stress erythropoiesis in hemolytic anemia model^35^, or in this study of a genetic model (*plcg1*^−/−^) to overcome the defect in HSPC formation. Taken together, our results provide a foundation to dissect potential novel mechanisms induced by ginger, and the future study of signaling interaction of Bmp-Notch-NO pathways to promote hematopoiesis.

## Methods

### Zebrafish husbandry

Zebrafish AB wildtype and transgenic lines *tg(flk1:GFP); tg(fli1:EGFP) y1* allele and *plcg1 y10* allele have been described previously^3,36,55,56^. Identified *tg(fli1:EGFP)* adult fish carriers of the heterozygous *plcg1*^+/−^ mutation are used and embryos are staged and maintained according to standard protocols^57,58^ and NCCU IACUC guidelines. “*plcg1*^−/−^ mutant” refers to homozygous (^−/−^) embryos, whereas “WT siblings” are phenotypically wildtype siblings that are heterozygous (^+/−^) or homozygous (^+/+^) embryos. WT refers to phenotypically wildtype non-transgenic embryos of AB strain. Zebrafish embryos are incubated in 0.3X Danieau’s solution (19.3 mM NaCl, 0.23 mM KCl, 0.13 mM MgSO_4_, 0.2 mM Ca(NO_3_)_2_, 1.7 mM HEPES, pH 7.4) at 28.5 °C; phenylthiourea (PTU, 30 µg/ml) is added around 8 hpf (75% epiboly stage) to inhibit pigment formation. Following exposure to ginger and/or pharmacological inhibitors as described, embryos are washed, dechorionated, and anaesthetized before imaging or fixing in 4% paraformaldehyde (PFA).

### Treatment of embryos with ginger and small-molecule inhibitors

Ginger and 10-gingerol preparation were described previously^35^. Ginger extract (5 µg/ml) and 10-gingerol (2 µg/ml) are diluted in 0.3X Danieau/PTU. All inhibitors are tested in a broad range of concentrations in 0.3X Danieau/PTU. LY411575 (γ-secretase inhibitor that blocks Notch activation), DMH1 (dorsomorphin analogue; highly selective Bmp inhibitor), SB505124 (inhibitor of TGF-β type I receptors ALK4/5/7), and L-NAME (Nos inhibitor) were obtained from Sigma-Aldrich; L-NMMA (Nos inhibitor) is obtained from Calbiochem. c-PTIO (NO scavenger) and SNAP (NO donor) are obtained from Molecular Probes.

### Whole-mount *in situ* hybridization

For experiments using *plcg1* mutants, embryos are sorted under a fluorescent stereomicroscope at 1dpf and distributed into two dishes: homozygous mutants (^−/−^) *versus* wildtype (WT) siblings (^+/−; +/+^), according to phenotype. Paraformaldehyde-fixed embryos at indicated stages are processed for *in situ* hybridization as described in Leung *et al*.^59^. The following antisense RNA probes are used: *scl*^60^, *runx1*^14^, *myb*^14^, *efnb2a*^3^, *notch3*^61^, *flt4*^61^, *bmp2b*^62^, *bmp7a*^63^, *pu.1*^14^, *gata1*^64^, *mpo*^14^, *lcp1*^65^, and *cd45*^65^. A minimum of 30 *plcg1*^−/−^ mutants embryos and 100 WT siblings per condition are used and 2–3 independent experiments are conducted per analysis. Pictures of zebrafish embryos are acquired using a Nikon SMZ1500 stereomicroscope with digital color camera DXM1200c (Nikon) and associated NIS-Element AR software.

### NO labeling *in vivo*

Control and treated embryos are incubated for 2 hours at 28.5 °C in the dark in their culture medium containing 5 µM DAF-FM-DA (Diaminofluorescein-FM diacetate, Molecular-Probes), then washed thoroughly and imaged using a fluorescence microscope. For NO scavenger c-PTIO, treatments are performed for only 30 minutes prior to NO labeling. For positive control, NO donor SNAP (10–50 µM) are applied to bud stage (end of gastrulation around 10 hpf) onward until analysis, whereas SNAP treatment at 250 µM, 2dpf embryos are incubated for 1 hr, which are previously loaded with DAF-FM-DA.

### Fluorescence microscopy

Real time imaging of embryos is performed using a MVX10 Macro-View Fluorescence Microscope (Olympus) with C9300-221 high-speed digital CCD camera (Hamamatsu). Picture acquisition parameters are kept constant to allow direct comparisons. Raw data are analyzed using MetaMorph Basic software (Olympus). Green fluorescence vasculature of *tg(fli1:GFP)* and NO staining are pseudo color images using MetaMorph.

### Ethical approval

All experimental protocols and procedures are approved by and conformed to the guidelines of the Animal Care and Use Committee of North Carolina Central University (Durham, NC). (NCCU IACUC Protocol # TCL-07-14-2008).

## Electronic supplementary material

Supplementary Information

## Data Availability

All data generated or analyzed during this study are included in this published article and supplementary figures.
