# Supplementary material for: Rescue of hematopoietic stem/progenitor cells formation in plcg1 zebrafish mutant
Source: Sci Rep. 2019 Jan 21;9:244. doi: 10.1038/s41598-018-36338-8 (PMC6341084; doi:10.1038/s41598-018-36338-8)

Supplementary Information

Rescue of hematopoietic stem/progenitor cells formation in *plcg1* zebrafish mutant.

Karine F. Ferri-Lagneau, Jamil Haider, Shengmin Sang and TinChung Leung

Supplementary figure legends

Supplemental Figure S1. Whole-mount *in situ* hybridization of the vein marker *fms*-related tyrosine kinase 4 (*flt4*) in 1dpf embryos. Scale bar = 250µm.

Supplemental Figure S2. Ginger promotes various blood lineage markers. A: Erythroid (*gata.1)*, early myeloid *(pu.1*), neutrophil *(mpo)* and myeloid (*lcp1*) lineage markers are evaluated by whole-mount *in situ* at indicated stages. **B:** The lymphoid (pan-leukocyte *cd45* marker) cell fate is also promoted by ginger. Arrows indicate the scattered lymphoid cells over the yolk area. Scale bars = 250µm.

Supplemental Figure S3. Like SNAP, sodium nitrite ($NaNO_2$) can induce NO levels, but has no effect on the CVP. A-C: Live images of the NO staining by DAF-FM-DA (**A-B**) and the vasculature in *tg(fli1:GFP)* (**C**) in 2dpf embryos. Scale bars = 250µm.

Supplemental Figure S4. The NO-promoting effect of ginger *in vivo* is dependent on Bmp signaling pathway, but independent of Notch activation. A: Notch inhibition (LY411575) does not prevent the increase of NO levels induced by ginger at 2dpf. **B-C:** Inhibition of Bmp

signaling pathway (DMH1) decreases the production of NO in the notochord at 2dpf (**B**), whereas it reduces the elevated NO levels induced by ginger throughout the body at 3dpf (**C**). White line indicates the junction of two composite images of the same embryo in (**C**). Scale bars = 250μm.

Supplemental Figure S1

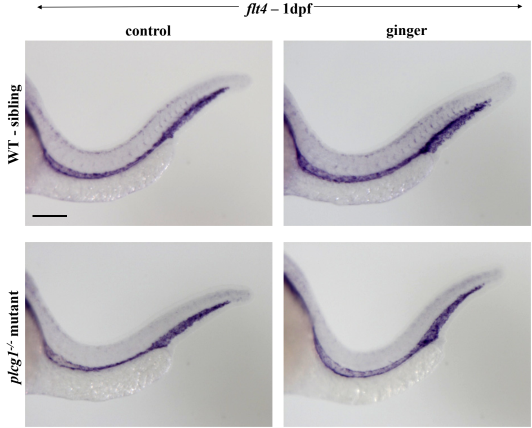

Supplemental Figure S2

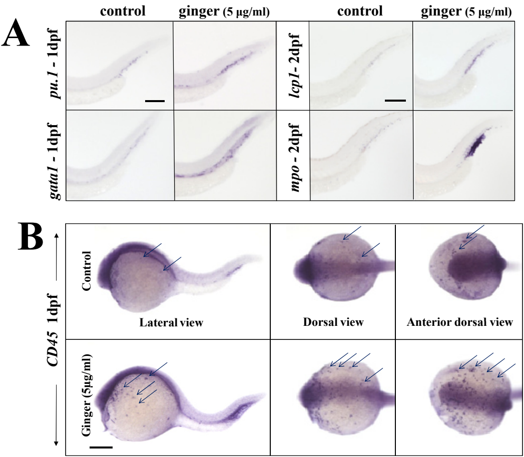

Supplemental Figure S3

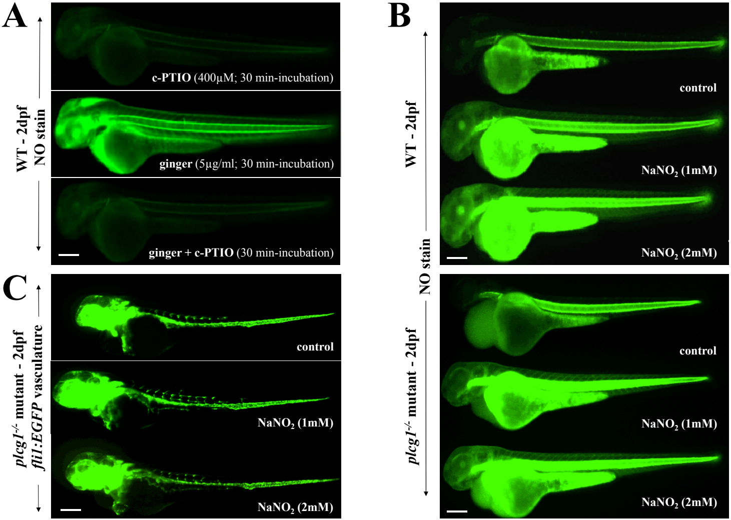

Supplemental Figure S4

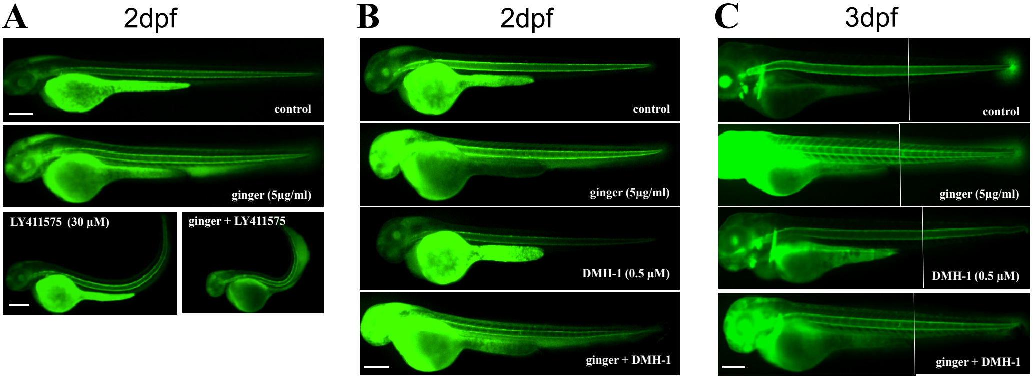

Supplement: Supplementary file 1 — Supplementary Information [file 41598_2018_36338_MOESM1_ESM.pdf]
